# Supplementary material for: Evolution of Gestational Diabetes Mellitus across Continents in 21st Century
Source: Int J Environ Res Public Health. 2022 Nov 28;19(23):15804. doi: 10.3390/ijerph192315804 (PMC9738915; doi:10.3390/ijerph192315804)
Supplement: Supplementary file 1 [file ijerph-19-15804-s001.zip › ijerph-1974626-supplementary.pdf]

Table S1. **Screening criteria according to countries and continents.**

Country	Screening Tests/Guidelines
Australia	ADS 1991, ADIPS98, IADPSG
New Zealand	NZSSD 1998, ADIPS98, IADPSG, NZMOH 2014
The United States of America	ADA, IADPSG/WHO 2013
Mexico	Not found
Canada	DC
Brazil	IADPSG/WHO 2013
Argentina	ALAD 2007, IADPSG/WHO 2013
Chile	The Ministry of Health of Chile 2015
Ecuador	IADPSG/WHO 2013
Colombia	IADPSG/WHO 2013
Peru	IADPSG/WHO 2013
Guyana	IADPSG/WHO 2013
Uruguay	IADPSG/WHO 2013
Trinidad and Tobago	IADPSG/WHO 2013
Paraguay, Suriname, Bolivia, Venezuela	Not found
Poland	IADPSG/WHO 2013
Spain	IADPSG/WHO 2013
Portugal	IADPSG/WHO 2013
France	IADPSG/WHO 2013
Italy	The Italian Public Health Authority criteria, IADPSG/WHO 2013
Germany	IADPSG/WHO 2013
Greece	IADPSG/WHO 2013
Switzerland	IADPSG
Austria	IADSPG
Czech Republic/Czechia	Irish criteria from 2010, French criteria from 2010, and Dutch from 2010, IADSPG, NICE 2015
Belgium	The NICE , IADPSG, Carpenter and Coustan
Netherlands	WHO 1999, IADPSG/WHO 2013,
United Kindgdon	WHO 1999, Scottish SIGN, AIDPSG/WHO 2013, NICE 2015,
Ireland	IADPSG/WHO 2013
Hungary	IADPSG/WHO 2013
Romania	IADPSG/WHO 2013
Iceland	IADPSG/WHO 2013
Denmark	O'Sullivan and Mahan, IADPSG/WHO 2013
Norway	WHO 1999; IADPSG/WHO 2013 and the Norwegian guidelines
Sweden	IADPSG/WHO 2013
Finland	Finnish Current Care Guidelines, The NICE, IADPSG
Slovenia	Carpenter and Coustan, IADPSG
Croatia	WHO 1999, IADPSG, The NICE
Serbia	ADA, IADPSG
Macedonia/ Northern Macedonia	IADPSG
Bosnia and Herzegovina	WHO 1999
Albania	The Albanian Diabetes Association
Estonia	IADPSG
Lithuania	WHO 1999, IADPSG
Cyprus	NDDG
Malta	IADPSG, WHO 2006, IADPSG/WHO 2013
Bulgaria	ADA, IADPSG/WHO 2013
Slovakia, Ukraine, Belarus, Latvia, San Marino, Liechtenstein, Monaco, Luxembourg, Moldova, Andorra, Georgia, Armenia, Azerbaijan	Not found
Oman	Oman self-defined guidelines

Bahrain	NDDG, ADA
Turkey	NDDG, IADPSG/China MOH /ADA2012/ WHO 2013/ADA 2014, C-C ACOG
UAE	C-C ACOG
Kuwait	Kuwait self-report GDM
Turkmenistan	Turkmenistan study-self defined guidelines
Yeman	WHO 1998, ADA 2002
Qatar	ADA 2004/WHO 2006, IADPSG/China MOH/ ADA2012/WHO 2013/ADA 2014,
Saudi Arabia	IADPSG,China MOH, ADA 2007, ADA 2011, ADA2012, WHO 2013,ADA 2014, C-C ACOG,
Iran	Kuwait self-report GDM, IADPSG,China MOH, ADA2012, WHO 2013, ADA 2014, C-C ACOG
Pakistan	WHO 1980, ADA 2004
Israel	C-C ACOG
Nepal	C-C ACOG, ADA 2007
Bangladesh	Bangladesh study-self defined guidelines, WHO 1999
Sri Lanka	WHO 1980, WHO 1985, WHO 1994, DIPSI, ADA 2007, IADPSG, China MOH, ADA2012, WHO 2013, ADA 2014,
India	WHO 1985, WHO 1994, WHO 1998 and ADA 2002, Kuwait self-report GDM, IADPSG, China MOH, ADA2012, WHO 2013, ADA 2014, DIPSI, C-C ACOG
Malaysia	Malaysia MOE, NICE, WHO 1985, WHO 1994, DIPSI, WHO 1999
Vietnam	IADPSG, China MOH, WHO 2013, ADA2012, ADA 2014
Singapore	ADA 2007 ,IADPSG, China MOH, ADA2012, WHO 2013, ADA 2014,
South Korea	IADPSG, China MOH, ADA 2012, WHO 2013, ADA 2014, C-C ACOG
Japan	Japan diabetes society, IADPSG, China MOH, ADA 2012, WHO 2013, ADA 2014
China	WHO 1999, IADPSG, China MOH, ADA 2012, WHO 2013, ADA 2014, C-C ACOG

Legend: WHO- World Health Organization, ADA - American Diabetes Association, IADPSG - International Asso-ciation of Diabetes and Pregnancy Study Groups, ALAD - the Latin American Diabetes Association, NDDG - The National Diabetes Data Group, Scottish SIGN - The Scottish Intercollegiate Guidelines Network, NICE - National Institute for Health and Care Excellence, MOH - Ministry of Health, DIPSI - Diabetes In Pregnancy Study Groups of India, C-C ACOG - The American Congress of Obstetricians and Gynecologists, UAE –United Arab Emirates, DC - Canadian Diabetes Association
